# Multi-TGDR, a multi-class regularization method, identifies the metabolic profiles of hepatocellular carcinoma and cirrhosis infected with hepatitis B or hepatitis C virus

**DOI:** 10.1186/1471-2105-15-97

**Published:** 2014-04-04

**Authors:** Suyan Tian, Howard H Chang, Chi Wang, Jing Jiang, Xiaomei Wang, Junqi Niu

**Affiliations:** 1 Division of Clinical Epidemiology, First Hospital of the Jilin University, 71Xinmin Street, Changchun, Jilin 130021, China; 2 Department of Hepatology, First Hospital of the Jilin University, 71Xinmin Street, Changchun, Jilin 130021, China; 3 Department of Biostatistics and Bioinformatics, Rollins School of Public Health, Emory University, 1518 Clifton Road NE, Atlanta, GA 30322, USA; 4 Department of Biostatistics and Markey Cancer Center, University of Kentucky, 800 Rose St., Lexington, KY 40536, USA

**Keywords:** Threshold gradient descent regularization (TGDR), Multi-class classification, Metabolic profile, Hepatocellular carcinoma (HCC), Feature selection, Metabolomics, Omics data

## Abstract

**Background:**

Over the last decade, metabolomics has evolved into a mainstream enterprise utilized by many laboratories globally. Like other “omics” data, metabolomics data has the characteristics of a smaller sample size compared to the number of features evaluated. Thus the selection of an optimal subset of features with a supervised classifier is imperative. We extended an existing feature selection algorithm, threshold gradient descent regularization (TGDR), to handle multi-class classification of “omics” data, and proposed two such extensions referred to as multi-TGDR. Both multi-TGDR frameworks were used to analyze a metabolomics dataset that compares the metabolic profiles of hepatocellular carcinoma (HCC) infected with hepatitis B (HBV) or C virus (HCV) with that of cirrhosis induced by HBV/HCV infection; the goal was to improve early-stage diagnosis of HCC.

**Results:**

We applied two multi-TGDR frameworks to the HCC metabolomics data that determined TGDR thresholds either *globally* across classes, or *locally* for each class. Multi-TGDR global model selected 45 metabolites with a 0% misclassification rate (the error rate on the training data) and had a 3.82% 5-fold cross-validation (CV-5) predictive error rate. Multi-TGDR local selected 48 metabolites with a 0% misclassification rate and a 5.34% CV-5 error rate.

**Conclusions:**

One important advantage of multi-TGDR local is that it allows inference for determining which feature is related specifically to the class/classes. Thus, we recommend multi-TGDR local be used because it has similar predictive performance and requires the same computing time as multi-TGDR global, but may provide class-specific inference.

## Background

Feature selection algorithms, which select a subset of the most relevant features for the underlying data mining tasks, are commonly used in combination with classifier construction to analyze “omics” data or data with high-dimensional input variables. The benefits of feature selection include minimizing model over-fitting, improved predictive performance, and computational efficiency. It may also provide insights on potential targets that relate to the fundamental differences among different classes or subtypes of a biological process [1]. Threshold Gradient Descent Regularization (TGDR) [2], one such algorithms, has been explored and implemented by us [3-5] extensively because it possesses several key advantages, as described in our previous paper [4].

Currently, multi-class classification, where an observation needs to be categorized into more than two classes, has attracted increasing attention in the statistics and bioinformatics literatures [6-10]. Its popularity may be attributed to the fact that multi-class classification is commonly encountered in real-world applications. For example, multiple classes can represent different tumor types or different responses to a therapy. According to Li et al. [6], multi-class classification can be roughly divided into two types. One type includes classification algorithms that can be directly extended to handle multi-class cases, and the other type includes algorithms that arise from the decomposition of multi-class problems into a series of binary ones.

While a series of binary TGDRs can be easily constructed to accomplish multi-class classification, it is more desirable to extend TGDR directly to the multi-class cases since this approach results in a substantial decrease of the number of classifiers being trained. The major technical difficulty associated with such extension of TGDR involves defining an overall threshold for a feature across different classes, which is not addressed in the original TGDR framework [2,11]. In our previous work [4], we introduced one approach, referred to as multi-TGDR, for determining the threshold function. We applied the proposed multi-TGDR framework to two real-world data conducted on the Affymetrix HG-U133 Plus 2 platform and demonstrated that multi-TGDR was superior in terms of predictive accuracy and parsimony compared to its binary counterparts (i.e., one-versus-another schema). In this paper, we propose a more general method to determine the threshold function, which allows the threshold function to be class-specific.

Metabolomics is the “…systematic study of the unique chemical fingerprints that specific cellular processes leave behind” [12]. Over the last decade, metabolomics has evolved into a mainstream scientific approach practiced by many laboratories globally. The information conveyed in metabolomics data can provide insight for a variety of applications such as biomarker identification, clinical toxicology, and drug discovery and development [13]. Like other “omics” data, metabolomics data typically has the characteristics of a smaller sample size compared to the number of features (usually hundreds of metabolites after peak alignment). Therefore, it is crucial to implement feature selection. However, metabolomics data analysis is less standardized compared to other “omics” data analysis (e.g., microarray and Next-Generation Sequencing [NGS]) due to its complexity. Consequently, many of the existing feature selection algorithms have not been explored and implemented in metabolomics data analyses. Only a few algorithms have been proposed to specifically analyze mass spectrometry (MS) data [14]. Reviews on feature selection algorithms that may be used in metabolomics data analyses have been reported [1,15].

*Partial Least Square-Discrimination Analysis* (PLS-DA) is a very popular multivariate analysis tool, which is commonly used in metabolomics data analyses to identify informative metabolites for many distinct purposes, such as the diagnosis or prognosis of a disease [16-19]. Notably, the success of the stand-alone software SIMCAP (http://www.umetrics.com) boosts the prevalence applications of PLS-DA in metabolomics data analyses. As a supervised method, PLS-DA rotates the *Principal Component Analysis* (PCA) components by using the class membership information to achieve a better separation between the classes of samples. Similar to PCA, the results from PLS-DA are based on some linear combinations of all metabolites or at least the linear combinations of the selected metabolites by naively leaving out the metabolites with small variable influence on the projection (VIP, which is a weighted sum of PLS loadings). This approach not only lacks readily biological interpretation, but also does not provide valid statistics that can be used to evaluate its predictive performance. To obtain such statistics, an extra classifier is desirable in PLS-DA. For example, the study by Student and Fujarewicz [10] obtained the accuracy of PLS-DA by implementing an additional support vector machine (SVM) classifier. Furthermore, absence of predictive rules in PLS-DA makes the results of PLS-DA less practical. This is because in clinical practice, physicians would prefer to a score (e.g., posterior probabilities) to quantify a patient’s status. Therefore, an explicit predictive rule is essential for metabolomics to become a diagnostic tool.

In this paper, we investigate the use of two multi-TGDR approaches to analyze mass spectrometry metabolomics data. Hepatocellular carcinoma (HCC) is the most common type of liver cancer. Most cases of HCC are secondary to either a viral hepatitis (hepatitis B or C) or cirrhosis [20]. Currently, the gold standards for diagnosis (e.g., ultrasonography and alpha-fetoprotein [AFP]) have been reported to lack satisfactory sensitivity and specificity for identifying HCC at early stages [21,22]. Since metabolomics can monitor the changes in small molecular comprehensively and provide insight on metabolic deregulations systematically [23,24], researchers are employing metabolomics techniques to elucidate the difference between HCC and cirrhosis [19]. The identification of metabolic profiles for HCC/cirrhosis infected with HBV or HVC may help discriminate between HCC/cirrhosis/normal classes and achieve accurate diagnosis of HCC at early stages. Moreover, the analyses presented in this paper also provide motivation for developing feature selection algorithms specifically for metabolomics data, and for the applications of existing algorithms to metabolomics data.

## Methods

### The experimental data

The study included 30 patients with cirrhotic liver disease (22 infected with HBV and 8 with HCV, respectively), 70 patient with HCC (39 with HBV infection and 31 with HCV infection), and 31 healthy volunteers recruited in the metabolic profiling study. All of them provided the written informed consent, and the ethics committee of the Jilin University approved upon this study. Detailed descriptions on the study design, experimental procedures, and LC-MS metabolomics data collections were reported in [19].

### Pre-processing procedures

Raw data were imported into Databridge (Waters, U.K.) for data format transformation. The resulting NeTCDF files were imported into XCMS software for the peak extraction and alignment. Then the peaks including 384 metabolites (indexed by the combination of m/z and retention time, and their corresponding peak intensities) and 131 samples were exported to an Excel file. The peak intensity values were log transformed so that the distribution of the transformed intensity values for each metabolite was approximately normal. Zeros (corresponding to no peaks) in peak intensity, were replaced by a nominal value (i.e., 0.01) before log transformation, to avoid the creation of missing values. Several other values for replacing zero values (i.e., 0.001, 0.005, 0.02, 0.05) were examined to evaluate if different nominal values would affect the results, and no difference was found. Finally, peak intensity values were further centralized and normalized to have a mean of 0 and a variance of 1. The resulting matrix was used in the two multi-TGDR frameworks for the classification analysis.

Compounds identification was achieved by comparing the accurate mass of compounds from the Human Metabolome Database: HMDB version 3 (http://www.hmdb.ca).

### Methods

Here, we omit the description of binary TGDR. Interested readers may refer to the original papers [2,11] for the detailed descriptions on binary TGDR. We present two multi-class TGDR frameworks with emphasis on the specific modifications made on the overall threshold functions to handle the multi-class problem.

#### ***Extension to multi-class classification***

In the multi-class cases, we have a set of C-1 binary variables Y_ci_ , which are the indicators for class c on subject *i* (*i* = 1,…,n, here n is the total number of subjects) i.e., Y_ci_ is equal 1 if the *i*^*th*^ subject belongs to class c and zero otherwise. *C* is the number of classes (C ≥ 3) and X_1_,…,X_n_ represents the feature values of one specific subject. Notably, X_i_ is a vector of length G and thus X is an n×G matrix with X_ij_ for the corresponding intensity values of feature *j (j = 1,…,G)* on subject *i*. The log-likelihood function is defined as,

(1) $$\begin{array}{ll} R\left(\beta \right)=\overset{n}{\underset{i=1}{\sum }} & \left(\overset{C-1}{\underset{c=1}{\sum }}Y_{\mathrm{ci}}\left(\beta_{c0}+\beta_{c}{X_{i}}^{T}\right)-\text{log}\right)\\ & \left(\left(1+\overset{C-1}{\underset{c=1}{\sum }}\text{exp}\left(\beta_{c0}+\beta_{c}{X_{i}}^{T}\right)\right)\right) \end{array}$$

β_c0_s (c = 1,…,C-1) are unknown intercepts which are not subject to regularization. β_c_ = (β_c1_,…, β_cG_) are the corresponding coefficients for the expression values of metabolites under consideration. In an ‘omics’ experiment, most of those betas are assumed to be zeros, implying the corresponding features are non-informative in explaining the difference across different classes. In the multi-class cases, the threshold functions of every feature (i.e., metabolites in our application) in TGDR need to be redefined across classes. In previous work [4], we proposed an extension of TGDR as described below.

#### ***Method 1***

Denote Δ*v* as the small positive increment (e.g., 0.01) in ordinary gradient descent search and *v*_*k*_ = k×Δ*v* as the index for the point along the parameter path after *k* steps. Let β(*v*_*k*_) denote the parameter estimate of β corresponding to *v*_*k*_. For a fixed threshold 0 *≤τ≤* 1, our proposed TGDR algorithm for multi-class cases is given as follows:

1. Initialize β(0)=0 and *v*_*0*_=0.

2. With current estimate β, compute the negative gradient matrix *g*(*v*) = - ∂*R*(*β*)/∂*β* with its (c,*j)*^*th*^ component as *g*_*cj*_*(v)*.

3. a) Let *f*_*c*_(*v*) represent the threshold vector of size G for class c (c=1,..,C-1), with its *j*^*th*^ component calculated as

$$f_{\mathrm{cj}}\left(v\right)=I\left(|g_{\mathrm{cj}}\left(v\right)|\geq \tau \times \underset{l\in \beta_{c}}{\text{max}\left(\left|g_{\mathrm{cl}}\left(v\right)\right|\right)}\right)\forall j\in \beta_{c}$$

b) Then, the *j*^*th*^-feature specific threshold function was defined as

$$f_{j}\left(v\right)=\underset{c}{\text{max}}\left(f_{\mathrm{cj}}\right)$$

4. Update β(v+Δv) = β(v) - Δv×g(v)×f(v) and update *v* by *v*+Δv , where the *(c, j)*^*th*^ component of the product of *f* and *g* is *g*_*cj*_(*v*) × *f*_*j*_(*v*).

5. Steps 2-4 are iterated *K* times. The number of iteration *K* is determined by cross validation.

As in binary TGDR, all metabolites are assumed to be non-informative at the initial stage. Parameters τ and k are the tuning parameters, and thus jointly determine the property of the estimated coefficients, including the selection of features and their corresponding magnitudes. τ can be regarded as a threshold because it determines how βs would be updated in each iteration. Two extreme cases include: if τ=0, all coefficients are nonzero for all values of k; and if τ=1, the multi-TGDR increases in the direction of one (if the gradient of the intercept term has the largest absolute value) or two covariates in each iteration. Consequently, the non-zero coefficients are few at the early iterations. With increasing k, increasing number of βs would deviate from zeros until all of them would eventually enter the model. Both τ and K are determined by using cross-validations [25].

In this framework, when one feature is selected in one comparison, it will appear in the rest comparisons even though it may not be informative in those comparisons. This is analogous to the multivariate regression model setting, where the same set of covariates is used for each response even though some of them may not be statistically significant. Alternatively, we may choose to force small estimated coefficients into zeros in the last step. Then, the set of selected features for each comparison becomes different. This framework is referred to as *multi-TGDR global* herein.

On the other hand, one may argue why not set *f*_j_ as the minimum of *f*_cj_s instead of their maximum. So if, there is no update until one feature has large enough gradients for all C-1 comparison. Therefore, only features which are informative in all comparisons will be chosen, resulting in an optimal feature set that is used to classify all classes simultaneously. This is in conflict with the hypothesis that a good feature set consists of those highly correlated with a class but uncorrelated with other classes, which had been confirmed by Hall [26]. Moreover, the performance of such determination has been proved to be generally less favorable than that of one-versus-another or one-versus-the rest binary ensembles [10].

#### ***Method 2***

**Figure 1:**
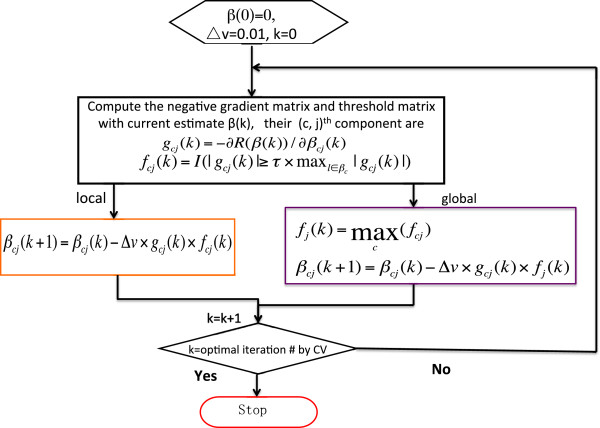
**The flowchart of multi-TGDR.** Global: multi-TGDR global; local: multi-TGDR local.

Instead of having an overall threshold function for j^th^ feature, a c^th^-class specific threshold function for the feature is used to select features. This modification corresponds to the step 3a in the multi-TGDR global framework. Thus, a feature is not necessarily selected in other comparisons when it is updated in one comparison. As a result, different sets of selected features are associated with different classes. This framework is herein referred to as *multi-TGDR local*. Figure 1 shows the flowcharts of multi-TGDR global and multi-TGDR local, and pinpoints the difference between two frameworks.

In the above two frameworks, we treat τ as a uniform tuning parameter across classes, which can certainly be relaxed so that τ may take different values for each class, allowing different degrees of regularization for different comparisons. However, for the “omics” data where the number of features is much larger than the number of samples, in our experience τ=1 tends to give the most reasonable results. Firstly, it has the harshest threshold, resulting in the smallest set of selected features. Secondly, the predictive performance may be improved by discarding those non-informative or redundant features.

#### ***Bagging and brier score***

Bagging [27] procedure was used to discard the possible noise from a single run of multi-TGDR, so that a better model parsimony can be warranted. The benefits of bagging include but are not limited to: avoidance of over-fitting, improvement on prediction, and manageable experimental verification. In many applications, e.g., [10], bootstrap resampling/bagging is mainly used to evaluate the stability of a classifier.

Besides the traditionally used confusion matrix and misclassification rate, the generalized brier score (GBS) [7] was also calculated to evaluate the predictive performance of two multi-TGDR frameworks by absorbing the extra information provided by the estimated posterior probabilities. Additional details on trimming performed on both bagging and brier score for multi-class classifications were discussed in a previous study [4].

#### ***Statistical language and packages***

The statistical analysis was carried out in the R language version 2.15 (http://www.r-project.org), R codes for multi-TGDR are available upon request.

## Results and discussion

### Synthesized data

In order to study the empirical performance of both multi-TGDR frameworks, we used the real values for metabolites of the HCC/cirrhosis data (384 metabolites and 131 samples) but assigned the class membership according to pre-determined logit functions *f*. Specifically, the logit functions for class 2 and 3, having class 1 as reference, were given by following relationship for two synthesized datasets,

#### ***Simulation 1***

$$\begin{array}{l} f_{2\mathrm{vs}1}=-0.1X_{1}-0.8X_{2}-0.9X_{3}+2X_{4}+1.2X_{5},\\ f_{3\mathrm{vs}1}=0.4X_{1}-0.5X_{2}-0.8X_{3}+1.7X_{4}-1.5X_{5} \end{array}$$

where the logits for class 2 and 3 depend only on features X_1_ ~ X_5_, but differ in the direction and magnitudes of the association.

#### ***Simulation 2***

$$\begin{array}{l} f_{2\mathrm{vs}1}=-0.1X_{1}+2X_{4}+1.2X_{5},\\ f_{3\mathrm{vs}1}=1.5X_{2}-0.8X_{3} \end{array}$$

where the logits for class 2 and 3 are two function with different parameters in the second simulation.

By this means, the true relevant features (i.e., X_1_*X*_2_ X_3_ X_4_ X_5_) are known and performance comparison can be made between multi-TGDRs and PLS-DA. Here, PLS-DA analysis was carried out in the software of SIMCA-P + version 12.0 (http://www.umetrics.com). A feature was eliminated unless it had VIP values larger than 1 in either of the first two PLS components. The results were given in Table 1.

**Table 1:** The comparison between Multi-TGDR frameworks and PLS-DA using simulated data

	**# metabolites**	**Error on the data (%)**	**GBS**	**5-fold CV Error (%)**
**A. Simulation 1**				
Multi-TGDR: global No Bagging	105	0.76	0.0100	12.21
Global + Bagging (freq > 30%)	35	10.69	0.0773	12.21
local No Bagging	54 (14, 46)^1^	2.29	0.0301	14.50
Local + Bagging (freq > 40%)	24 (14, 15)	8.40	0.0539	12.98
PLS-DA + Naïve Bayes as a classifier	89	14.50	0.1313	19.84
**B. Simulation 2**				
Multi-TGDR: global No Bagging	110	0	0.0165	11.45
Global + Bagging (freq > 50%)	21	3.82	0.0237	7.63
local No Bagging	106(12, 95)	0	0.0067	9.16
Local + Bagging (freq > 40%)	25(9, 18)	3.82	0.0254	8.40
PLS-DA + Naïve Bayes as a classifier	97	6.87	0.1556	16.03

A. The performance of multi-TGDR frameworks and PLS-DA on the first simulated data. B. The performance of multi-TGDR and PLS-DA on the second simulated data.

^1^(No.1, No.2): No.1 represents the number of metabolites selected in the first comparison (class 2 versus class 1) by multi-TGDR local. No.2 represents the number of metabolites selected in the second comparison (class 3 versus class 1).

In summary, the true relevant features were successfully identified by all methods. The predictive performance of both multi-TGDR frameworks was superior to that of PLS-DA. Even after bagging, the final models for both multi-TGDRs include substantially more features than the true ones, which might indicate more improvement in the multi-TGDR frameworks and other relevant algorithms may exist.

### Real data

**Figure 2:**
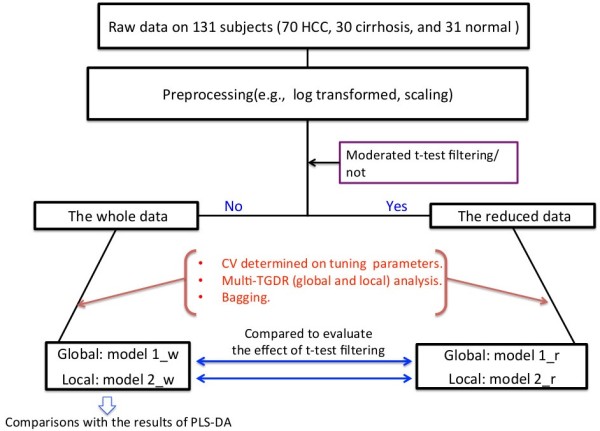
The schema of the study.

A metabolomics study was conducted with the objectives of identifying potentially important differential metabolites related to HCC pathogenesis and early diagnosis, and thus providing an explicit predictive rule that can aid a physician’s diagnosis on HCC and cirrhosis. There were 131 subjects (70 with HCC, 30 with cirrhosis, and 31 normal controls, respectively) and 384 metabolites in this study. Additional details on this motivating study have been presented in [19]. Figure 2 outlines the schema of this study.

#### ***Performance of multi-TGDR***

**Figure 3:**
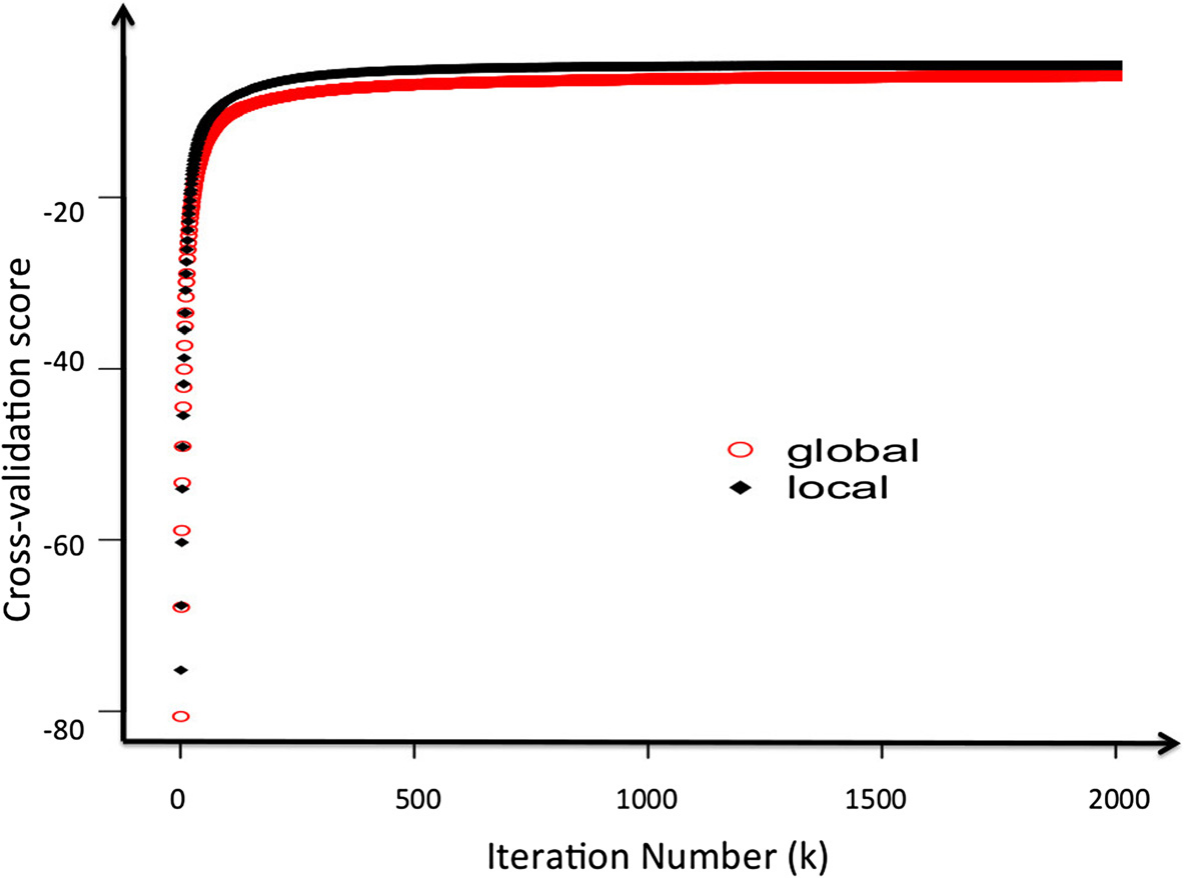
**The comparison between cross validation (CV)-determined tuning parameter k (the iteration number) in both multi-TGDR frameworks.** Global: multi-TGDR global; local: multi-TGDR local.

In Figure 3, cross-validation scores showed minimal change, especially after k > 500 for both frameworks. So the final iteration number K in both Multi-TGDR global and Multi-TGDR local was chosen as 500. Table 2 presents the results of the two multi-TGDR approaches. Multi-TGDR global selected 45 metabolites with a 0% misclassification rate and a 3.82% 5-fold cross-validation predictive error rate. With the cutoff of bagging frequency fixed at 40%, 30 metabolites were retained in the final model (Model 1_w), which had a 0% misclassification rate and a slight improvement on GBS. On the other hand, Multi-TGDR local selected 48 metabolites with a 0% misclassification rate and a 5.34% CV-5 error rate. After applying Bagging, 29 were identified in the final model (Model 2_w) with a slight decrement in GBS. Interestingly, the metabolites in model 1_w and model 2_w are almost the same (25 overlapped). Model 1_w identified 5 extra metabolites and model 2 _w identified 4 such metabolites. Table 3 presented those overlaps and those extra metabolites identified by specific multi-TGDR framework.

**Table 2:** The predictive performance of Multi-TGDR frameworks and PLS-DA

	**# metabolites**	**Error on the data (%)**	**GBS**	**5-fold CV Error (%)**
**A.** (without filtering)				
Multi-TGDR global No Bagging	45	0	4.24e-05	3.82
Bagging (freq > 40%)	30	0	3.68e-05	3.82
Multi-TGDR local No Bagging	48	0	7.57e-05	5.34
Bagging (freq > 40%)	29	0	5.97e-04	6.11
**B.** (after moderated *t*-test filtering)				
Multi-TGDR global No Bagging	42	0	1.03e-04	4.58
Bagging (freq > 25%)	37	0	1.13e-04	4.58
Bagging (freq > 40%)	26	0	3.58e-04	4.58
Multi-TGDR local No Bagging	42	0	6.18e-04	6.11
Bagging (freq > 25%)	38	0	6.87e-04	5.34
Bagging (freq > 40%)	25	0	2.24e-03	6.11
**C.** the performance of PLS-DA on the whole data				
Naïve Bayes as the extra classifier	42	4.58	4.63e-02	7.63

A. The performance of multi-TGDR frameworks on the whole data: without moderated *t*-test filtering. B. The performance of multi-TGDR frameworks on the reduced data: with *t*-test filtering and 72 metabolites were filtered out. C. The performance of PLS-DA with naïve Bayes as the classifier. 42 metabolites selected by original analysis in Zhou’s study ref. [19] were used.

Note: For the reduced data, the optimal cutoff of bagging frequencies is 25%. However, in order to make a fair comparison with the results from the whole data, we analyzed the reduced data with bagging frequencies as 40% as well.

**Table 3:** The selected metabolites by both multi-TGDR frameworks (the results of model 1_w and model 2_w)

**type**	**mz**	**RT (min)**	**β_Cirrhosis**	**β_HCC**	**β_Cirrhosis**	**β_HCC**	**Metabolites**
			**Multi-TGDR global**		**Multi-TGDR local**		
**All**	191.04	0.64	0.6521	-0.6011	0.7437	-0.4971	Beta-Lactose
	240.08	14.79	-0.2734	0.2258	-0.2842	0.0267	1,1′-Ethylidenebistryptophan or 1-aminopyrene
	582.24	22.42	-0.4816	0.2596	-0.4854	0.1428	Glutaminyl-Methionine
**Common**	91.36	8.47	0.1937	-0.9035	0	-0.4716	Unknown
	100.32	1.16	0.1965	-0.6931	0	-0.7513	Unknown*
	101.32	1.16	0.1209	-0.4617	0	-0.4617	Unknown
	139.1	8.65	0.1969	-0.4631	0	-0.4631	Phosphorylcholine
	218.08	0.89	0.2614	-0.7598	0	-0.9121	Pregnenolone sulfate
	255.96	1.07	0.041	0.2284	0	0.3122	Lsoxanthopterin
	256.25	19.08	-0.358	0.9489	0	0.9761	Palmitic amide*
	279.08	9.4	0.034	-0.3269	0	-0.4019	Homocarnosine
	361.18	19.64	-0.0196	0.0538	0	0.1811	Unknown
	540.51	23.17	0.0211	0.2882	0	0.2898	Unknown
	599.25	9.78	0.5919	3.8635	0	4.1545	Unknown
	239.14	14.47	-0.2198	0.0995	-0.3349	0	Phosphatidic acid
	289.21	7.24	-0.1335	-0.0097	-0.1347	0	Neurosporene
	356.37	15.91	0.3772	-0.0728	0.2259	0	Unknown
	374.38	15.45	0.5133	0.0856	0.2179	0	Unknown
	375.39	15.46	1.4191	0.1173	1.3393	0	Cholestanetriol or Unknown
	402.42	17.55	0.2209	0.0424	0.4634	0	16(S)-hydroxy-18-oxo-18-CoA-LTE4
	585.27	9.09	1.435	-0.1864	1.7132	0	Conjugated bilirubin*
	587.27	9.09	0.3402	0.1137	0.2372	0	Conjugated bilirubin*
	592.37	6.42	0.3208	-0.0508	0.5229	0	Unknown
	633.25	10.31	-0.7011	0.4138	-0.7187	0	Unknown
	652.41	4.19	1.2071	0.4634	1.4441	0	Ganglioside GM3 (d18:1/24:0) or Unknown
**Global**	181.08	8.6	-0.1244	0.0115	0	0	Alpha-Ketooctanoic acid
	277.17	10.69	-0.1585	0.1796	0	0	Phosphatidylinositol or Lithocholate 3-O-glucuronide
	312.37	18.9	-9.00E-04	-0.0819	0	0	Unknown
	315.19	8.7	-0.1527	0.0978	0	0	3-Oxohexadecanoic acid
	608.38	3.97	0.1159	0.0187	0	0	Unknown
**Local**	159	0.62	0	0	-0.158	0.1842	Glycolaldehyde
	330.35	15.37	0	0	0.1697	0	Unknown*
	634.26	10.31	0	0	-0.0769	0	Indoleacetyl glutamine
	810.62	29.87	0	0	0	-0.1817	SM(d18:1/18:0)

The normal controls serve as the reference. All: non-zeros in both comparisons and both versions; Common: selected by both versions, but being zero in one comparison by local; global: selected only by multi-TGDR global version; local: selected only by multi-TGDR local version. Model 1_w: the results of multi-TGDR global after bagging (BF > 40%); Model2_w: the results of multi-TGDR local after bagging (BF > 40%).

Note: *the overlaps with the metabolites selected by PLS-DA.

#### ***Comparison with PLS-DA analysis***

The data had also been analyzed using PLS-DA [19]. There, the potential markers were chosen based on the loading plot of PLS-DA, then evaluated by VIP of the first two components in PLS-DA and further confirmed by t-tests. We compared the selected metabolites by the original analysis with the resulting metabolites from Multi-TGDR frameworks (using the whole data on which the original PLS-DA was conducted), there are only 4 overlaps between multi-TGDR global and PLS-DA, and 5 overlaps between multi-TGDR local and PLS-DA, respectively (indicated as * in Table 3).

In order to compare results obtained from PLS-DA and those from Multi-TGDR, we used the 42 metabolites selected by PLS-DA (as shown on Table 2 in [19]) and considered a naïve Bayes model as a classifier to calculate the posterior probabilities in PLS-DA. The performance of PLS-DA was shown in Table 2. In summary, the metabolites selected by Multi-TGDR have a better predictive performance than those by PLS-DA.

#### ***Evaluation on the effect of pre-processing filtering***

**Figure 4:**
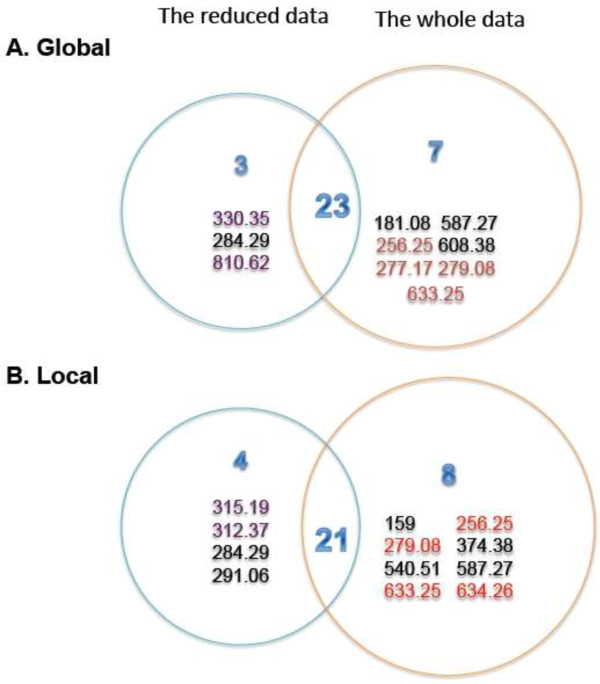
**The comparison of the selected metabolites by multi-TGDR frameworks on the whole data and on the reduced data (BF > 40% for both data). A**. Venn-diagram for multi-TGDR global. **B**. Venn-diagram for multi-TGDR local. The whole data: without moderated *t*-test filtering. The reduced data: with *t*-test filtering and 72 metabolites were filtered out. The metabolites (indexed by m/z values) in red represent those filtered out by moderated t-tests. The metabolites (indexed by m/z values) in purple represent those selected by multi-TGDR framework on the whole data analysis, but excluded by bagging.

Moderated t-tests using limma [28] were conducted to identify the differential metabolites between HCC/cirrhosis versus normal to examine the effects of pre-filtering. The cutoff for the false discovery rate (FDR) was chosen as 0.05. There were 94 down-regulated and 104 up-regulated metabolites in the comparison of cirrhosis to normal, and 63 down-regulated and 186 up-regulated metabolites for HCC to normal. In total, 302 unique differentially expressed metabolites were identified by those t-tests. Only 4 metabolites were missing from the final classifier models (i.e., model 1_w and 2_w). We then reran both multi-TGDRs with those 302 differentially expressed metabolites. The corresponding results were shown in Table 2B and Figure 4. From them, we can see the performance of both multi-TGDR on the filtered data decreased but was not substantial. To conclude, pre-filtering may save considerable computational time with marginal impact on predictive performance.

## Conclusions

Metabolites selected by multi-TGDR may provide biological insight in HCC/cirrhosis. According to the description of those selected metabolites given by the HMDB, some interesting observations were gained. First, furoic acid is a metabolite produced by furfural via oxidation. Furfural is a confirmed animal carcinogen with unknown relevance to humans, and it has been suggested as a substance that produces hepatic cirrhosis [29,30]. Here, both multi-TGDR versions selected furoic acid, while the coefficients of both comparisons (i.e., HCC versus normal, cirrhosis versus normal) are in opposite directions. A significant decrease of isoxanthopterin has been identified in cancer patients [31], however, the multi-TGDR results show an increase instead. It is well known that careful control of the participants’ intake before a metabolomics experiment is difficult. With that in mind, many of the HCC subjects may have received therapeutic treatments that might increase the level of isoxanthopterin, with residual levels present despite strict diet and intake control during the metabolomics study. In addition, over-dosage of interventions for cancer patients, especially in a developing country like China is possible. Thus the accumulation of isoxanthopterin in HCC patients is possible as a result of long-term over-dosage of relevant drugs. Meanwhile, we don’t exclude the possibility that HCC has its own unique characteristics in terms of isoxanthopterin and is consequently different from other cancer types. Further investigation on the biological explanation of those selected metabolites is definitely warranted. Here, our focus is to present the multi-TGDR frameworks and to demonstrate their applications in metabolomics.

With the aids of a feature selection algorithm like multi-TGDR (an algorithm can provide an explicit predictive rule and compute the posterior probabilities of the class membership), it is possible to design a diagnostic kit to examine the selected metabolites in a clinic setting with higher sensitivity and specificity. This kit would allow discrimination between HCC developed from HCV/HBV infections apart from cirrhosis with HCV/HBV infections, which is highly desirable and of scientific importance. One limitation of our application is that since the proportion of diseased persons in an observational study may not reflect disease prevalence in the population, care must be taken in both model construction and evaluation. To ensure a multi-TGDR model can correctly classify persons in the general population, one approach is to obtain weights based on the ratio between the proportion of diseased persons in the population and that in the study. A comprehensive investigation of these issues is the focus of our future research.

Two extensions of TGDR are proposed here for multi-class classification problems. By training only one classifier, we specifically address sub-optimality associated with dividing multi-class classification into individual binary pairs. The performance of multi-TGDR global has been shown to be excellent by us previously [4] using simulated data and two microarray data sets. Compared to multi-TGDR global, multi-TGDR local has an almost identical predictive performance in the HCC metabolomics data (in both the simulated data and the real data). Additionally, we conducted extra simulations to verify the validity of multi-TGDR local and compared its performance with multi-TGDR global. The results (included in the Additional file 1: Supplementary materials) show that both multi-TGDR algorithms can identify the true relevant features and discard the irrelevant features. Identical predictive performances are also observed even in cases where some of features are highly correlated to the relevant features. Intuitively, we hypothesized that multi-TGDR global should perform better in cases where the classes share more similarity. This entails that the same set of features is shared across different classes, but the magnitudes of the association differ. This may correspond to different stages of a disease. In contrast, multi-TGDR local may be optimal in cases where no similarity of the classes is present. This entails that complete different sets are selected across different classes, which may represent different diseases. Interestingly, the results from the simulations don’t support this hypothesis. Finally, we also examined whether multi-TGDR local is associated with less computation time since it does not need to compute the overall threshold function *f*_*j*_(*v*). However, with our current experience on the simulations and real-world applications, we found the computational effort of these two approaches to be comparable. This may be due to the fact that compared to the computation of many gradients at each iteration, the computation of maximum on *f*_*cj*_(*v*) is negligible. One obvious advantage of multi-TGDR local is that it may provide us with information on which feature is related to which class/classes.

To conclude, we recommend the use of the multi-TGDR frameworks for multi-class classifications on “omics” data because they have excellent predictive capacity. The researchers may choose to run both or either of the multi-TGDR frameworks based on their research hypotheses and data type.

## Abbreviations

TGDR: Threshold gradient descent regularization; multi-TGDR global and local: Threshold gradient descent regularization for multiple classes (global: version 1 and local: version 2); HCC: Hepatocellular carcinoma; CV-X: X fold cross validation; BF: Bagging frequency; HMDB: Human Metabolome Database; GBS: Generalized brier score; PCA: Principal Component Analysis; PLS-DA: Partial Least Square-Discrimination Analysis.

## Competing interests

The authors declare that they have no competing interests.

## Authors’ contributions

Conceived and designed the study: ST JQN. Analyzed the data: ST CW. Interpreted data analysis and results: HHC ST CW XMW JJ. Contributed materials/analysis tools: XMW JQN JJ. Wrote the paper: ST HHC JJ JQN CW. All authors reviewed and approved the final manuscript.

## Supplementary Material

Additional file 1 Supplementary materials. Click here for file
