# Supplementary material for: Multi-TGDR, a multi-class regularization method, identifies the metabolic profiles of hepatocellular carcinoma and cirrhosis infected with hepatitis B or hepatitis C virus
Source: BMC Bioinformatics. 2014 Apr 4;15:97. doi: 10.1186/1471-2105-15-97 (PMC4234477; doi:10.1186/1471-2105-15-97)
Supplement: Additional file 1 — Supplementary materials. [file 1471-2105-15-97-S1.doc]

## Additional file 1: Supplementary Materials

Feature Generation

We assumed a total of 100 features. Feature values were generated from 100 standard normal (mean=0, variance=1) random variables (i.e., X_1_ ,…, X_100_) for 100 subjects.

For each simulation, 50 datasets were generated according to the analyzed by the proposed multi-TGDR frameworks. We considered two scenarios describing the dependence between features. In the first scenario, the 100 features assumed to be independent from one another, and it is referred to as iid scenario. In the second scenario, we set cor(X_1_, X_5_) =cor(X_3_, X_7_)=0.5 and cor(X_2_, X_6_) =cor(X_4_, X_8_)=-0.5, and it is referred to as the correlated scenario.

Class Generation

We assumed there are three classes arising from the features. Here, we considered two extreme cases. First, the sets of relevant features in different comparisons are exactly the same; second there is no overlap between the sets of relevant features in separate comparisons. Intuitively, one may argue multi-TGDR global should work better in case 1 while local fits better in case 2, however, simulations show both frameworks provide approximately identical results in these two extreme cases.

***Extreme case I***

Example 1 (strong signal): Using one group as the reference and the logit functions for the other two groups are as follows,

$\begin{matrix} f_{1}=2X_{1}+1.2X_{2}-0.8X_{3} \\ f_{2}=1.7X_{1}-1.5X_{2}-X_{3} \end{matrix}$.

Here, the intercepts in both logit functions are fixed at zero, so the simulated samples tend to be balanced (i.e., with almost equal sizes in each class).

Example 2 (moderate signal): Then we simulated another set of simulated data using smaller coefficients to represent moderate signals.

$$\begin{matrix} f_{1}=-0.1X_{1}-0.8X_{2}-0.9X_{3} \\ f_{2}=0.4X_{1}-0.5X_{2}-0.8X_{3} \end{matrix}$$

***Extreme case II***

Example 3 (strong signal): Still, using one group as the reference, we assumed the logit functions for the other two groups are as followings,

$\begin{matrix} f_{1}=2X_{1}-0.8X_{3} \\ f_{2}=1.5X_{2}+X_{4} \end{matrix}$ .

Example 4 (moderate signal): the coefficients of the logit functions in this example are smaller compared to those in example 3.

.

Simulation Results

It is observed that more than 80% times the true relevant features be selected by multi-TGDR frameworks in the iid scenario. As criticized by Wang et al [1], lack of parsimony is an obvious disadvantage of TGDR algorithms. As an extension, multi-TGDR also inherits this shortcoming. Our simulations show that by using Bagging procedure, parsimony was improved.

Both the size of final selected feature and the predictive errors for correlated scenario were almost the same compared with the iid scenario. Overall, multi-TGDR always successfully selected the relevant features and had good predictive performance. Nevertheless, it is observed that when the signals were weaker, multi-TGDR frameworks tended to select more redundant features into the final models, resulting in more false positives. However, the probability of selecting the informative ones is high unless the signal is very week. For example, even when coefficient=0.1, the BF is still above 40%.

Bagging procedure improved on parsimony and predictive performance. Thus, it is highly recommended to combine bagging with any TGDR algorithm, even though bagging is very computationally intensive. Multi-TGDR local outperformed multi-TGDR global in terms of parsimony, consequently identifying less false positives.

References:

1. Wang Z, Chang YI, Ying Z, Zhu L, Yang Y**: A parsimonious threshold-independent protein feature selection method through the area under receiver operating characteristic curv**e*. Bioinformati*cs 2007**,** 23:2788–94.

Supplementary Table 1: The results for extreme case I (example I)

| A: Multi-TGDR global | | | | | | | | | |
| --- | --- | --- | --- | --- | --- | --- | --- | --- | --- |
|  | β_1_≠0 (%)  Average BF (%) | | β_2_≠0 (%)  BF(%) | | | β_3_≠0 (%)  BF(%) | | genes (#) | Average predictive error (%) |
| Iid case:  no bagging | 100  100 | | 100  100 | | | 100  99.90 | | 35.08 | 18.94 |
| BF>40% | --- | | --- | | | --- | | 31.20 | 18.86 |
| BF>80% | --- | | --- | | | --- | | 5.66 | 16.38 |
| Correlated case no bagging | 100  **100** | | 100  **100** | | | 100  **99.94** | | 37.5 | 18.18 |
| BF>40% |  | |  | | |  | | 32.74 | 18.00 |
| BF>80% |  | |  | | |  | | 5.68 | 15.50 |
| B. Multi-TGDR local | | | | | | | | | |
| Comparison | 1^st^ | 2^nd^ | | 1^st^ | 2^nd^ | 1^st^ | 2^nd^ |  |  |
| iid case no bagging | 100  **99.24** | 100  **96.64** | | 100  **97.56** | 100  **100** | 88  **76.90** | 100  **98.02** | 19.32 | 19.86 |
| BF>40% | --- | --- | | --- | --- | --- | --- | 13.28 | 19.98 |
| BF>80% | --- | --- | | --- | --- | --- | --- | 3.72 | 18.80 |
| Correlated case no bagging | 100  **99.98** | 100  **97.92** | | 100  **97.20** | 100  **100** | 96  **81.68** | 100  **97.84** | 22.14 | 17.52 |
| BF>40% |  |  | |  |  |  |  | 14.80 | 17.30 |
| BF>80% |  |  | |  |  |  |  | 4.02 | 17.28 |

Supplementary Table 2: The results for extreme case I (example 2)

| A: Multi-TGDR global | | | | | | | | | |
| --- | --- | --- | --- | --- | --- | --- | --- | --- | --- |
|  | β_1_≠0 (%)  Average BF (%) | | β_2_≠0 (%)  BF(%) | | | β_3_≠0 (%)  BF(%) | | genes (#) | Average predictive error (%) |
| Iid case:  no bagging | 100  100 | | 100  100 | | | 100  100 | | 42.3 | 15.86 |
| BF>40% | --- | | --- | | | --- | | 31.96 | 15.44 |
| BF>80% | --- | | --- | | | --- | | 7.46 | 11.28 |
| Correlated case no bagging | 100  **100** | | 100  **100** | | | 100  **100** | | 43.86 | 16.4 |
| BF>40% |  | |  | | |  | | 32.86 | 16.28 |
| BF>80% |  | |  | | |  | | 7.36 | 13.72 |
| B. Multi-TGDR local | | | | | | | | | |
| Comparison | 1^st^ | 2^nd^ | | 1^st^ | 2^nd^ | 1^st^ | 2^nd^ |  |  |
| iid case no bagging | 80  57.04 | 98  93.44 | | 100  100 | 100  99.52 | 100  99.96 | 100  100 | 42.72 | 16.50 |
| BF>40% | --- | --- | | --- | --- | --- | --- | 26.42 | 15.58 |
| BF>80% | --- | --- | | --- | --- | --- | --- | 5.54 | 13.24 |
| Correlated case no bagging | 66  44.04 | 100  96.28 | | 100  100 | 100  99.48 | 100  99.88 | 100  99.84 | 43.34 | 16.08 |
| BF>40% | --- | --- | | --- | --- | --- | --- | 28.12 | 16.12 |
| BF>80% | --- | --- | | --- | --- | --- | --- | 5.98 | 12.96 |

Supplementary Table 3: The results for extreme case II (example 3)

| A: Multi-TGDR global | | | | | | | | | | |
| --- | --- | --- | --- | --- | --- | --- | --- | --- | --- | --- |
|  | β_1_≠0 (%)  BF (%) | | β_2_≠0 (%)  BF(%) | | β_3_≠0 (%)  BF(%) | | β_4_≠0 (%)  BF(%) | | genes (#) | error (%) |
| Iid case:  no bagging | 100  100 | | 100  100 | | 100  97.18 | | 100  **99.82** | | 31.84 | 13.38 |
| BF>40% | --- | | --- | | --- | | --- | | 23.36 | 13.32 |
| BF>80% | --- | | --- | | --- | | --- | | 5.7 | 10.54 |
| Correlated: no bagging | 100  **100** | | 100  **100** | | 100  **95.88** | | 100  **99.96** | | 33.38 | 12.32 |
| BF>40% |  | |  | |  | |  | | 25.48 | 11.84 |
| BF>80% |  | |  | |  | |  | | 5.9 | 8.74 |
| B. Multi-TGDR local | | | | | | | | | | |
|  | 1^st^ | 2^nd^ | 1^st^ | 2^nd^ | 1^st^ | 2^nd^ | **1^st^** | **2^nd^** |  |  |
| iid case:  no bagging | 100  **100** | 8  **12.24** | 10  **11.58** | 100  **100** | 94  **88.50** | 14  **11.48** | 8  **13.26** | 100  **97.78** | 19.64 | 13.34 |
| BF>40% | --- | --- | --- | --- | --- | --- |  |  | 13.36 | 13.96 |
| BF>80% | --- | --- | --- | --- | --- | --- |  |  | 4.38 | 12.94 |
| Correlated:  no bagging | 100  **100** | 6  **10.08** | 8  **8.98** | 100  **100** | 98  **90.70** | 22  **15.18** | 10  **9.86** | 100  **97.40** | 22.62 | 12.56 |
| BF>40% |  |  |  |  |  |  |  |  | 15.06 | 12.48 |
| BF>80% |  |  |  |  |  |  |  |  | 4.62 | 11.88 |

Supplementary Table 4: The results for extreme case II (example 4)

| A: Multi-TGDR global | | | | | | | | | | |
| --- | --- | --- | --- | --- | --- | --- | --- | --- | --- | --- |
|  | β_1_≠0 (%)  BF (%) | | β_2_≠0 (%)  BF(%) | | β_3_≠0 (%)  BF(%) | | β_4_≠0 (%)  BF(%) | | genes (#) | error (%) |
| Iid case:  no bagging | 100  94.6 | | 100  100 | | 100  100 | | 100  99.72 | | 38.08 | 13.28 |
| BF>40% |  | |  | |  | |  | | 29.08 | 12.86 |
| BF>80% |  | |  | |  | |  | | 6.82 | 11.06 |
| Correlated: no bagging | 100  92.20 | | 100  100 | | 100  100 | | **100**  99.56 | | 36.84 | 14.84 |
| BF>40% | --- | | --- | | --- | | --- | | 27.62 | 14.44 |
| BF>80% | --- | | --- | | --- | | --- | | 6.66 | 10.86 |
| B. Multi-TGDR local | | | | | | | | | | |
|  | 1^st^ | 2^nd^ | 1^st^ | 2^nd^ | 1^st^ | 2^nd^ | **1^st^** | **2^nd^** |  |  |
| iid case:  no bagging | **98**  93.76 | **8**  9.08 | **38**  29.84 | **100**  100 | **100**  100 | **6**  7.4 | **40**  27.08 | **100**  99.32 | 33.8 | 16.26 |
| BF>40% | --- | --- | --- | --- | --- | --- | --- | --- | 21.64 | 16.18 |
| BF>80% | --- | --- | --- | --- | --- | --- | --- | --- | 5.4 | 14.62 |
| Correlated:  no bagging | 98  **90.60** | 10  **9.52** | 56  **34.00** | 100  **100** | 100  **100** | 0  **7.28** | 38  **32.64** | 100  **99.52** | 33.18 | 15.74 |
| BF>40% | --- | --- | --- | --- | --- | --- | --- | --- | 21.52 | 15.52 |
| BF>80% | --- | --- | --- | --- | --- | --- | --- | --- | 5.52 | 13.52 |
